# Supplementary material for: Normalisation process theory and the implementation of a new glaucoma clinical pathway in hospital eye services: Perspectives of doctors, nurses and optometrists
Source: PLoS One. 2021 Aug 2;16(8):e0255564. doi: 10.1371/journal.pone.0255564 (PMC8328316; doi:10.1371/journal.pone.0255564)
Supplement: Attachment — Submitted filename: Reviewer Responses.docx [file pone.0255564.s001.docx]

| **Ref** | **Reviewer** | **Reviewer Comment** | **Author Response** |
| --- | --- | --- | --- |
| 001 | Editorial Board | Please review your reference list to ensure that it is complete and correct. If you have cited papers that have been retracted, please include the rationale for doing so in the manuscript text, or remove these references and replace them with relevant current references. Any changes to the reference list should be mentioned in the rebuttal letter that accompanies your revised manuscript. If you need to cite a retracted article, indicate the article’s retracted status in the References list and also include a citation and full reference for the retraction notice. | **We have added two further references based on the feedback points received below (specifically ref: 009). Aside from that, the only change within the references of the manuscript is the unblinding of a related article (reference no. 12).** |
| 002 | Editorial Board | Please ensure that your manuscript meets PLOS ONE's style requirements, including those for file naming. | **We believe the existing manuscript complies fully with PLOS ONE’s style requirements.** |
| 003 | Editorial Board | Please include captions for your Supporting Information files at the end of your manuscript, and update any in-text citations to match accordingly. | **N/A – No Supporting Information is provided.** |
| 004 | Editorial Board | We note that you have indicated that data from this study are available upon request. PLOS only allows data to be available upon request if there are legal or ethical restrictions on sharing data publicly. | **There are ethical issues associated with sharing the full data publicly given that these are extended qualitative interview transcripts gathered from a relatively small locality. There are concerns that, in spite of being de-identified, staff may still be identifiable to those familiar with the study and other health care professionals should the full transcripts be made accessible more widely. This is due to the relatively small network of health boards involved in the sample, as well as the nature of the interviews themselves where aspects of practice that are potentially unique to certain settings are included for context. This was embedded into our initial ethical application and is therefore an ongoing requirement. Furthermore, participants provided their consent on the basis of their anonymity being maintained. Ethical opinion was provided by West Midlands – Black Country NHS Research Ethics Committee, Village Urban Resort Dudley, 2 Castlegate Park, Birmingham Road, Dudley, West Midlands, DY1 4TB.** |
| 005 | Reviewer 1 | Line 179- please clarify what does "independent of the health board" mean. | **This phrase is indicating that the researchers conducting the fieldwork were employed by a separate institution, e.g. Cardiff University, and were independent from the day-to-day activities of the health board in which they were performing the research. We have changed the phrase to be ‘independent from the clinical team’ and believe this should enhance the clarity for readers.** |
| 006 | Reviewer 1 | Line 191- Under clinical observations. Because it is not clear how many researchers were collecting the data it would be helpful to explain what is meant by "52 week-by-week." means. Is it 52 consecutive weeks? Or 52 field notes? Please state if these field notes are referencing the "wider observational data" in Line 187, are they the same thing? | **We agree that this would benefit from further clarity. As we have established the nature of the field notes in question in the previous two sentences, we have adjusted the wording here to be: “A total of 52 of these observational field notes were taken over the duration of the study covering the majority of research visits”** |
| 007 | Reviewer 1 | Line 209 "favourable ethical opinion" sounds odd, is this equivalent to ethical approval? | **Yes – it is exactly the same but is based on the terminology of the UK-based ethical committees. Given the international readership and audience of PLOS ONE, we have amended this to ‘ethical approval’ for the purposes of this article.** |
| 008 | Reviewer 1 | Line 233 "where data should sit" It is not clear what this phrase means. | **We have adapted this sentence to further enhance clarity. This now reads ‘where data fitted within the developing themes’.** |
| 009 | Reviewer 1 | Line 259 Staffing - what is the value of about mapping not having a staff to NPT constructs (in this case cognitive participation>initiation, enrolment), rather than just using a more straightforward implementation framework that would mention manpower/ staffing/ champions? This for me seems counter intuitive especially for someone more familiar with other implementation frameworks. Suggest to consider this as the NPT fit is discussed under Discussion>limitations. | **We have added some wordings in the Study Limitations section as suggested, including reference to frameworks where implementation issues related to staffing are more clearly established, such as through the processes of knowledge translation approaches and the use of change champions during implementation etc.** |
| 010 | Reviewer 1 | Line 315- It is hard to tell who these staff are in terms of having more formal knowledge of glaucoma, perhaps some indicator of why they are considered so should be included after their quotes e.g. OH 301 () | **We have provided one or two examples within the text to highlight what is intended by this phrase. There is a risk that by aligning these to specific quotes that we enable identification of participants so believe this to be a preferable solution.** |
| 011 | Reviewer 1 | Line 397 - This theme is quite relevant as the 4 hour + 4 week schedule clearly illustrates how elasticity and plasticity can be applied to analysing feasibility. However, I wonder if the CMGC is really just about these larger parameters which were of course quite easy to predict in terms of how they would affect feasibility. It would be interesting if authors could also illustrate more macro level examples of these concepts to show how smaller instructions in the CMGC were either elastic/ plastic or not. | **We feel that we have offered a thorough discussion of this topic and highlighted several macro level examples within the Discussion section (lines 448-495 and 513-532). Given the other changes we have made above, particularly in relation to ref: 009 in the limitations section, we believe this comment is adequately covered.** |
| 012 | Reviewer 1 | Line 420- " that the retention rate of patients for all procedures ran at 94.3%" Should clarify if this refers to CMGC and if so across the same period of observation as this study (ie was it a sister/ substudy)? | **We have clarified this in-text by confirming that the retention rate was for all CMGC study procedures.** |
